# A “Diabetes Acute Care Day” for medical students increases their knowledge and confidence of diabetes care: a pilot study

**DOI:** 10.1186/s12909-016-0600-x

**Published:** 2016-03-09

**Authors:** A. W. MacEwen, D. M. Carty, A McConnachie, G. A. McKay, J. G. Boyle

**Affiliations:** Department of Endocrinology and Diabetes, Medical Block, Glasgow Royal Infirmary, 84 Castle Street, Glasgow, Scotland; Undergraduate Medical School, University of Glasgow, Glasgow, Scotland; Robertson Centre for Biostatistics, University of Glasgow, Glasgow, Scotland

**Keywords:** Diabetes, Care, Knowledge, Confidence, Medical and students

## Abstract

**Background:**

Evidence suggests that junior doctors lack the confidence and skills to manage acute/inpatient diabetes. We investigated the impact of the introduction of a “Diabetes Acute Care Day” on undergraduate medical students’ knowledge and confidence in acute/inpatient diabetes.

**Methods:**

Participants attended four short lectures on the basics of diabetes, diabetic emergencies, inpatient diabetes management and peri-operative/procedure care followed by case-based learning tutorials on diabetic ketoacidosis (DKA), hyperosmolar hyperglycaemic state (HHS) and hypoglycaemia using capillary blood glucose charts to interpret and practice subsequent insulin prescription and adjustment. Participants were asked to complete multiple-choice questions and confidence questionnaires using a visual analogue score pre and post participation.

**Results:**

One hundred forty-four students completed the pre-course survey and 196 completed the post-course survey. Mean confidence using a visual analogue score increased in all areas with a mean at baseline of 46.9 mm rising to 71.2 mm post-participation (*p* < 0.001). The largest increases were in the management of HHS, patients on subcutaneous and intravenous insulin and perioperative/procedure care. The mean mark obtained in the pre-test multiple choice questions (MCQs) was 2.72 (27.2 %) and increased to 4.74 (47.4 %) on the post-score MCQs (*p* < 0.001). 56.9 % of participants answered all 10 pre-test MCQs with the mean number of questions answered = 4.71 rising to 82.0 % of students answered all ten questions and the mean number of questions answered = 9.56 in the post-test MCQs.

**Conclusions:**

An intensive “Diabetes Acute Care Day” consisting of themed live lectures and case-based learning tutorials is an effective way to increase medical students’ knowledge and confidence in acute/inpatient diabetes. Further development and evaluation of this educational intervention is required to assess the impact of on patient care in the clinical setting post graduation.

## Background

Approximately 10–20 % of hospital inpatients in the UK have diabetes and the majority of care for these patients is provided by Foundation year doctors early in postgraduate training, often out of hours and without specialist supervision [1]. Foundation year doctors have a central role in the delivery of inpatient diabetes care to national standards laid down by the national service framework for diabetes and require to have these competencies at the time of graduation [2].

Undergraduate medical curricula are failing to prepare new foundation year doctors to meet these standards [3]. In a recent NHS cross-sectional audit of inpatient diabetes care in over 200 UK hospitals 39.8 % of patients had at least one medication management, insulin or prescription error during their inpatient stay. After admission, 10.5 % developed severe hypoglycaemia and 0.5 % patients developed new diabetic ketoacidosis (DKA). Patients with medication errors had nearly twice the rate of severe hypoglycaemia [4]. The National Patient Safety Agency (NPSA) has received over 16,000 reports of insulin-related incidents and has now issued a rapid response report to improve prescribing and the administration of insulin [5].

The Trainees Own Perceptions of Delivery of Care (TOPDOC) Diabetes Study presented the views of existing postgraduate trainees in the UK on their self-reported confidence, current practice and training needs in diabetes [6]. This national survey of over 2000 UK doctors in training indicated a lack of confidence in diagnosing and managing all aspects of diabetes among trainee doctors. More than 70 % of trainees wanted more training in all aspects of diabetes care. Overall only 42 % of respondents felt that their undergraduate training had prepared them to make a diagnosis of diabetes, 49 % felt their undergraduate training had prepared them to treat diabetic emergencies and just 19 % concluded that their undergraduate training had prepared them to optimise the treatment of diabetes.

These findings are reinforced by a survey of Consultant Diabetologists affiliated with the University of Birmingham on the undergraduate teaching of diabetes and endocrinology which suggested that only 13 % of respondents felt that the knowledge of diabetes at the end of Year 5 was adequate [7]. The majority (73 %) of diabetologists felt that the current level of teaching on diabetes has left foundation year doctors ill-equipped to manage diabetes when they leave medical school with 87 % concluding that the development of an undergraduate curriculum for diabetes was essential. These results are less surprising when one considers that the Society for Endocrinology recently stated that many medical schools do not provide compulsory exposure to diabetes for all their students and curricula seldom include practical issues such as insulin types, dose adjustment, initiation and management of insulin infusions and peri-operative diabetes care [8].

We hypothesised that undergraduate medical student knowledge and confidence in acute/inpatient diabetes care was low in their final clinical year and that it could be improved by the delivery of a “Diabetes Acute Care Day”. Here we present the results of this pilot evaluation of the first “Diabetes Acute Care Day”.

## Methods

The University of Glasgow’s five year MBCHB programme follows a spiral curriculum over four phases and contains seven vertical themes including clinical skills, vocational and professional studies, health of populations and communities, pharmacology, clinical pharmacology and prescribing, anatomy and imaging and basic biomedical sciences. Phase 4 (Clinical years 1 and 2) students participate in five to ten week clinical attachments as well as attending academic days consisting of small group work and lectures designed to complement clinical attachments.

The study was approved by the ethics committee for non-clinical research involving human subjects (College of Medical, Veterinary and Life Science) at The University of Glasgow. 272 Clinical year 1 medical undergraduates were eligible to attend the ‘Diabetes Acute Care Day’. All potential participants were emailed an information sheet explaining the study one week in advance. All students attending the “Diabetes Acute Care Day” were provided with the information sheet, multiple-choice questions (MCQs) and self-reported confidence questionnaires using a visual analogue score. Participation of at least 110 medical undergraduates (40 %) was taken as a representative sample of the year. Participation was anonymous and submission of the completed form was taken as tacit consent to participate in the study. Participants were advised that they could withdraw at any point.

The intended learning objectives (ILOs) for the day included making a diagnosis of diabetes, diagnosis and management of diabetic ketoacidosis (DKA), diagnosis and management of hyperosmolar non-ketotic state (HHS), diagnosis and management of hypoglycaemia, diabetes in the inpatient setting (interpreting capillary blood glucose result charts, managing patients on subcutaneous (SC) insulin therapy, managing patients on intravenous (IV) insulin, prescribing IV fluids for patients with diabetes, managing patients on oral hypoglycaemic agents) and altering diabetes therapy in the peri-operative/procedure setting. Participants were made aware of the ILO’s in advance but no pre-reading material was provided.

Participants attended four 20–30 min live lectures on the basics of diabetes, diabetic emergencies, inpatient diabetes management and peri-operative/procedure care. This was delivered to the entire year group simultaneously. This was followed by case-based learning tutorials lasting 90 min in 24 groups of 12 students. There were 3 different cases on DKA, HHS and hypoglycaemia each with multi-stem questions. The case-based learning tutorials were concluded with 12 different capillary blood glucose charts to interpret and practice subsequent insulin prescription and adjustment. During small group work participants were encouraged to select appropriate management plans for each scenario including insulin doses, relevant instructions and advice about monitoring. Authentic insulin prescriptions charts and care pathways where appropriate. The teachers facilitated discussion.

Two hundred seventy-two fourth-year medical undergraduates were eligible to attend the ‘Diabetes Acute Care Day’. Participants were provided with an information sheet, multiple choice questions (MCQs) and self-reported confidence questionnaires using a visual analogue score and were given 20 min prior to starting the teaching and immediately following the last tutorial to complete them. The MCQs were formative, mapped to each of the 10 ILOs and designed to assess the knowledge required of a UK foundation trainee. The scores obtained in the pre-test served as the baseline data. Written answers for each scenario with explanations and further reading were made available to participants on completion of the post-course MCQs and questionnaire. 144 participants completed the pre-test MCQs and questionnaire and 196 participants completed the post-test MCQs and questionnaire. Responses were anonymous and so pre and post course results for individual participants were assessed but analysed as independent samples. The total participation time on the day was 240 min.

MCQs were marked out of ten. No response was taken as incorrect. Student self-rated confidence was measured using a 100 mm visual analogue score. For analysis of the study data, total MCQ scores were compared using a Wilcoxon-Mann–Whitney test. Individual MCQ scores were analysed using Fisher’s exact test and confidence scores were compared using a two-sample t-test. Data were analysed using SPSS Version 16 (SPSS Inc., Chicago, Il, USA) and Microsoft Excel 2007 (Microsoft Corporation, Redmond, Washington, USA). *P*-values less than 0.05 were considered as statistically significant.

## Results

**Fig. 1:**
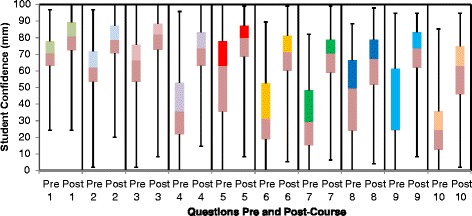
Student confidence pre and post Diabetes Acute Care Day

**Fig. 2:**
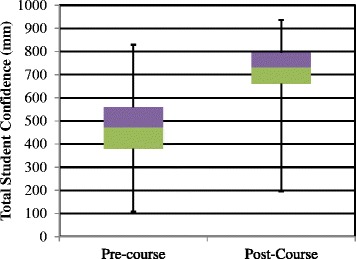
Total Student Confidence pre and post course

In total, 144 students (53 %) completed the pre-course confidence survey and 196 (72 %) the post-course confidence survey. Overall mean confidence was 46.9 mm at baseline rising to 71.2 mm. Baseline confidence was lowest in peri-operative diabetic care and management of patients on IV and s/c insulin. Baseline confidence was highest in diagnosis of diabetes and in diagnosis and management of DKA and hypoglycaemia. Confidence levels improved significantly in all ten questions (See Fig. 1) and overall (See Fig. 2) (*p* < 0.001). The largest increases were in the management of HHS, patient on s/c and iv insulin and perioperative/procedure care (See Table 1).

**Fig. 3:**
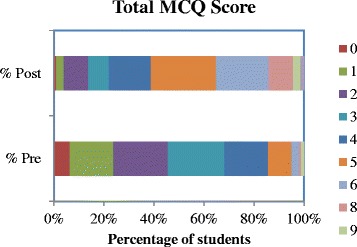
Percentage of students in each total score category (0–9) on pre and post course formative multiple-choice questions

**Fig. 4:**
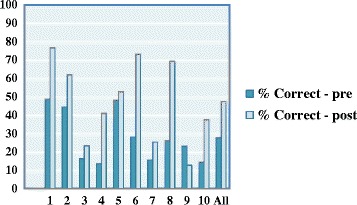
Multiple Choice Question (MCQ) Scores Pre and Post Acute Care Day

The mean mark obtained in the pre-test MCQs was 2.72 (27.2 %). Baseline knowledge was highest in diagnosing and managing DKA, managing hypoglycaemia and prescribing IV fluids for patients with diabetes. Baseline knowledge was lowest in managing patients on IV insulin, diagnosing and managing HHS, managing patients on oral hypoglycaemic agents and altering diabetes therapy prior to surgery. The MCQ score increased significantly to 4.74 (47.4 %) on the post-score MCQs (*p* < 0.001) (See Fig. 3). Only 91/160 (56.9 %) of students answered all 10 pre-test MCQs (mean number of questions answered 4.71) and this rose to 168/205 (82.0 %) of students answered all ten questions (mean number of questions answered 9.56 in the post-test MCQs). Scores improved significantly on the post-test MCQs for 7 out of 10 of the questions (See Fig. 4). The questions were mapped to the following ILOs: diagnosing and managing DKA, prescribing IV fluids for patients with diabetes managing patients on IV insulin, diagnosing and managing HHS, managing patients on oral hypoglycaemic agents and altering diabetes therapy prior to surgery. There was no significant change on questions 3 and 5 (ILO’s: managing patients on IV insulin and managing hypoglycaemia) and a decrease in score on question 9 (ILO: altering diabetes therapy prior to procedures). Response rates improved significantly on all questions in the post-test MCQs (See Table 2).

**Table 1:** Mean Confidence Scores Pre and Post Diabetes Acute Care Day (DKA – Diabetic ketoacidosis, IV – Intravenous, HHS – Hyperosmolar hyperglycaemic state, S/C – subcutaneous)

Question: How confident are you at:		Mean Confidence (mm)		Change in confidence (mm)	*p*
		Pre-Course	Post-course		
1	Making a diagnosis of diabetes?	68	77	+8	<0.001
2	Diagnosing and managing hypoglycaemia?	60	77	+17	<0.001
3	Diagnosing and managing DKA?	63	79	+16	<0.001
4	Diagnosing and managing HHS?	38	71	+33	<0.001
5	Interpreting capillary blood glucose result charts?	57	76	+19	<0.001
6	Managing patients on SC insulin therapy?	36	68	+33	<0.001
7	Managing patients on IV insulin?	33	68	+36	<0.001
8	Managing patients on oral hypoglycaemic agents?	45	64	+19	<0.001
9	Prescribing IV fluids for patients with diabetes?	44	71	+26	<0.001
10	Altering diabetes therapy prior to surgery/procedures?	26	59	+33	<0.001
	Overall Mean	46.9	71.2	+24.3	<0.001

**Table 2:** Change in Multiple Choice Question (MCQ) Score and Response Rate Pre and Post Diabetes Acute Care Day for each Intended Learning Objective (ILO) (DKA – Diabetic ketoacidosis, IV – Intravenous, HHS – Hyperosmolar hyperglycaemic state, S/C – subcutaneous)

MCQ	Intended Learning Objective(s)	Pre-test (*n* = 160)		Post-test (*n* = 205)		Change in score		Change in no response	
		Correct	Blank	Correct	Blank	%	*P*	%	*P*
1	Diagnosing DKA/Prescribing IV fluids for patients with diabetes	78 (48.8 %)	8 (5 %)	157 (76.6 %)	2 (0.9 %)	+27.8 %	<0.001	+4.0 %	0.024
2	Managing DKA	71 (44.4 %)	13 (8.1 %)	127 (62.0 %)	3 (1.5 %)	+17.6 %	0.001	+6.7 %	0.003
3	Managing patients on IV insulin	26 (16.3 %)	15 (9.4 %)	48 (23.4 %)	5 (9.4 %	+7.2 %	0.115	+6.9 %	0.005
4	Diagnosing and Managing HHS	22 (13.8 %)	23 (14.4 %)	84 (41.0 %)	11 (5.4 %)	+27.2 %	<0.001	+9 %	0.004
5	Managing Hypoglycaemia	77 (48.1 %)	23 (14.4 %)	108 (52.7 %)	3 (1.5 %)	+4.6 %	0.4	+12.9 %	<0.001
6	Managing patients on SC insulin therapy	45 (28.1 %)	41 (25.6 %)	150 (73.2 %)	8 (3.9 %)	+45.4 %	<0.001	+21.7 %	<0.001
7	Managing patients on oral hypoglycaemic agents	25 (15.6 %)	46 (28.8 %)	52 (25.4 %)	7 (3.4 %)	+9.7 %	<0.001	+25.3 %	<0.001
8	Interpreting capillary blood glucose result charts/Managing patients on SC insulin therapy	42 (26.3 %)	55 (34.4 %)	142 (69.3 %)	13 (6.3 %)	+43.0 %	<0.001	+28.0 %	<0.001
9	Altering diabetes therapy prior to procedures	37 (23.1 %)	97 (60.6 %)	26 (12.7 %)	20 (9.8 %)	−10.4 %	0.012	+29.6 %	<0.001
10	Altering diabetes therapy prior to surgery	23 (14.4 %)	97 (60.6 %)	77 (37.6 %)	19 (9.3 %)	+23.2 %	<0.001	+30.1 %	<0.001

## Discussion

To our knowledge this is the first study exploring an education intervention medical schools could use to prepare medical students to provide diabetes care in hospital. The results of the pilot study demonstrate that delivery of a “Diabetes Acute Care Day” during undergraduate medical education significantly increases medical students’ knowledge and confidence in diabetes care.

At baseline we found low overall mean participant confidence (46.9/100 mm) and this was reflected by the low number of questions answered in the pre-course MCQs with the mean number of questions answered at baseline being 4.71. This finding is similar to the findings in the TOPDOC diabetes study that post-graduates lack confidence in many aspects of inpatient diabetes care [6]. The greatest increase in confidence was seen in areas with the lowest baseline confidence and these areas included management of s/c and IV insulin, HHS and peri-operative/procedure care.

In addition to low baseline confidence levels we also found the baseline knowledge of acute/in-patient diabetes care was low with a mean MCQ score of 27.2 %. The data shows that MCQ scores improved most for questions related to interpretation of capillary blood glucose results charts and dose adjustment of SC insulin suggesting that the chosen format is a particularly effective approach to teaching students to prescribe insulin safely. This was also reflected by a greater increase in student confidence in managing patients on both IV and s/c insulin than in other areas such as diagnosing diabetes and diagnosing and managing hypoglycaemia and diabetic ketoacidosis.

There were two questions where there was no significant change in participant knowledge. One MCQ question related to the switching of a patient from IV to SC insulin in the management of DKA. In the MCQ the insulin regimen used was different to that used in case based learning scenario (multiple daily injections and twice daily biphasic regimen). This suggests that many students were unable to apply their knowledge in a different clinical scenario and had reached the fourth phase of Kolb’s experiential learning cycle by attempting to apply new knowledge to a similar scenario although the majority had done so incorrectly [8]. We completed Kolb’s cycle by providing detailed written answers and discussion to each of the case based learning scenarios and all the MCQ’s after the completion of the study. We hope that this should allow the students to reflect on their incorrect answers and apply the correct action when encountering a similar situation in the future [9]. In the second question we noted that knowledge of the current driving regulations for patients with hypoglycaemia was required to correctly answer this question. This reinforces the need to constructively align the intended learning objective to both the educational content and formative assessment of future days as we did not directly cover this area in either the live lectures or the case based learning scenarios [10].

There was also one question when knowledge actually decreased. This question related to altering diabetes therapy prior to a procedure. It is interesting to note that this is one of the areas where student confidence increased the most. This intended learning objective was covered in the final theme lecture but not in a case-based learning scenario. In our analysis of this unexpected finding we considered Bloom’s Taxonomy [11], which categorises various thinking processes required for learning into a hierarchy with each level being dependent on the ability to perform at the levels before it [11]. The ILO’s for the “Diabetes Acute Care Day” all used an active verb such as “managing” and thus can be categorised up and including Bloom’s fifth level of learning – Synthesis. Table 3 demonstrates how we could apply this taxonomy to the MCQ, which related to altering twice-daily biphasic insulin therapy in a patient with Type 2 diabetes prior to undergoing a colonoscopy. By applying this taxonomy we can begin to understand why a student may increase their level of confidence but at the same time fail to demonstrate that they have achieved this ILO in an assessment. Indeed, while the delivery of themed live lectures may increase levels one and two (“knowledge” and “comprehension”) the absence of an aligned case-based learning scenario with the chance to actively prescribe fails to give students the opportunity to achieve levels 3,4 and 5: “analysis”, “application” and “synthesis”.

**Table 3:** Blooms Taxonomy Applied to altering insulin therapy prior to a colonoscopy in a patient with Type 2 Diabetes

Level		Relevant Intended Leaning Outcome
1	Knowledge	Describe how different insulin preparations work.
2	Comprehension	Recognise potential causes of hypoglycaemia and hyperglycaemia in a patient with diabetes undergoing a procedure.
3	Application	Modify different insulin regimens to maintain euglycaemia in a patient with diabetes undergoing a procedure.
4	Analysis	Identify causes of decompensated diabetes in a patient with diabetes undergoing a procedure.
5	Synthesis	Formulate a management plan to maintain euglycaemia for a patient with diabetes undergoing a procedure.
6	Evaluation	Justify your chosen management plan.

Post-course mean MCQ score were still low at 47.4 % and alternative models of teaching should be considered. Further work includes plans to evolve the next ‘Diabetes Acute Care Day’ using the ‘flipped classroom’, a pedagogical model in which the traditional lecture and homework elements of a course are “reversed” or “flipped” [12, 13]. An advance in technology at the University of Glasgow has led to the opportunity to use blended learning initiatives (which combine classroom and online education). The goal of the Flipped Classroom would be to provide an opportunity for student’s to consume course related material at their own pace and on their own time prior to the actual day. It is hoped that when students arrive, they are more ready to discuss and apply this new knowledge thereby facilitating deep learning and the attainment of higher learning outcomes. This will involve the use of recorded video lectures, online quizzes, discussions boards with frequently asked questions for a period of 4 weeks before the day. The time previously allotted for live lectures will be replaced with student-centred active learning strategies to facilitate the acquisition of the desired higher order intended learning outcomes. This will involve the use of recorded video lectures before the day. The time previously allotted for live lectures will be replaced with active learning strategies to facilitate the acquisition of the desired higher order intended learning outcomes.

Given that the MCQ questions were designed around clinical scenarios that a foundation year doctor is likely to encounter in daily practice this suggests that further work is required to ensure graduates are competent in acute/inpatient diabetes care. The participants were in the final clinical year and therefore still have more training to complete, including a period of preparation for practice (junior doctor shadowing), before taking up their first post as a junior doctor. It is important to note that this evaluation was immediately after completion of the day. We therefore plan to assess the legacy effect by repeating a formative MCQ examination and confidence survey in the same cohort of students at graduation as well as comparing the results of acute diabetes/inpatient diabetes questions in the final examinations to previous cohorts of students who have not participated in a Diabetes Acute Care Day.

In this study we have used pre and post-course MCQs as a means of assessing student knowledge. It is, however, important to note that Miller’s framework of competency assessment states that tests of knowledge alone are insufficient to properly assess educational interventions or to predict performance in clinical practice [14, 15]. The inclusion of clinical based cases to the MCQ questions does allow students to demonstrate Miller’s second level of competence (“Knows How”) however the higher levels (“Shows How” and “Does”) cannot be assessed here [15]. Further work could include the addition of authentic clinical charts and pathways in the assessment of the day.

The evaluation was completed at the end of the day but as the participants were in the final clinical year they still have more training to complete. This would include self-directed learning based on the materials provided and a period of preparation for practice (junior doctor shadowing) before commencing their postgraduate training. We are therefore unable to evaluate the final impact on patient care (Kirkpatrick’s 4th level of evaluation). The legacy effect on the care of patients could be assessed by comparing performance in the clinical setting post-graduation with graduates from the previous academic year.

The authors acknowledge several limitations. The main limitation of this study is the lack of a control group with which to compare the intervention to. Moreover, the number of responses at the end of the day was higher than the beginning of the day. As participation was anonymous it was not possible to compete a paired analysis and this weakens the strength of the results. The use of a study identification number on the MCQ and confidence questionnaire booklet would facilitate this. Even when taking student attendance into account the response rate was less than 100 % and this could lead to a degree of selection bias. The response rate could be improved by summative rather than formative assessment. Curricular development is now underway so that the ILO’s from the ‘Diabetes Acute Care Day’ can be mapped to the final written and objective structured clinical examinations facilitating assessment on higher levels on Miller’s pyramid [15].

## Conclusions

An intensive “Diabetes Acute Care Day” consisting of themed live lectures and case-based learning tutorials is an effective way to increase medical students’ knowledge and confidence in acute/inpatient diabetes before graduation. Further development and evaluation of this educational intervention is required assess the impact on patient care in the clinical setting post graduation.
